# HDL, Atherosclerosis, and Emerging Therapies

**DOI:** 10.1155/2013/891403

**Published:** 2013-05-28

**Authors:** Anouar Hafiane, Jacques Genest

**Affiliations:** ^1^McGill University, Montreal, QC, Canada H3A 1A1; ^2^Faculty of Medicine, Center for Innovative Medicine, McGill University Health Center, Royal Victoria Hospital, McGill University, 687 Pine Avenue West, Montreal, QC, Canada H3A 1A1

## Abstract

This review aims to provide an overview on the properties of high-density lipoproteins (HDLs) and their cardioprotective effects. Emergent HDL therapies will be presented in the context of the current understanding of HDL function, metabolism, and protective antiatherosclerotic properties. The epidemiological association between levels of HDL-C or its major apolipoprotein (apoA-I) is strong, graded, and coherent across populations. HDL particles mediate cellular cholesterol efflux, have antioxidant properties, and modulate vascular inflammation and vasomotor function and thrombosis. A link of causality has been cast into doubt with Mendelian randomization data suggesting that genes causing HDL-C deficiency are not associated with increased cardiovascular risk, nor are genes associated with increased HDL-C, with a protective effect. Despite encouraging data from small studies, drugs that increase HDL-C levels have not shown an effect on major cardiovascular end-points in large-scale clinical trials. It is likely that the cholesterol mass within HDL particles is a poor biomarker of therapeutic efficacy. In the present review, we will focus on novel therapeutic avenues and potential biomarkers of HDL function. A better understanding of HDL antiatherogenic functions including reverse cholesterol transport, vascular protective and antioxidation effects will allow novel insight on novel, emergent therapies for cardiovascular prevention.

## 1. Introduction 

An increasing body of literature emphasizes the concept that HDL functionality, rather than the absolute cholesterol mass (HDL-C), may be a more accurate indicator for risk of developing atherosclerosis [1]. This hypothesis has led to investigation of HDL as both a biomarker for cardiovascular risk and a therapeutic target to be functionally modulated [2]. Epidemiological studies consistently demonstrate that low plasma level of HDL-C is associated with increased risk of CVD, but this epidemiological association has not translated into evidence that raising HDL-C prevents CVD. Atherosclerosis remains the leading cause of death in developed countries and is a major health concern worldwide. While LDL cholesterol (LDL-C) is clearly established as the major lipoprotein risk factor [3], the residual risk in large-scale clinical trials raises concern that other lipoprotein fractions may be causal in this residual risk. Increasingly, questions have been raised around the hypothesis that raising HDL-C pharmacologically is necessary beneficial. In this regard, after the recent failure of the drugs torcetrapib, dalcetrapib, and niacin [4, 5] that raise HDL-C, attention is focusing on specific HDL subfractions and on biomarkers of HDL function (reflecting its pleiotropic effects) as potential therapeutic targets for cardiovascular protection [6–8]. Such studies have reinforced the need for validated assays of HDL function rather than static measurement of HDL-C. A variety of HDL/apoA-I-based therapies are currently under investigation. This review summarizes the biology of HDL and the importance of reverse cholesterol transport process in lipid-modifying therapy and discusses the novel therapeutic agents to raise HDL.

## 2. Definition: HDL

HDL is the smallest and densest of plasma lipoproteins. HDL isolated by ultracentrifugation is defined as the lipoprotein with density in the range 1.063–1.21 g/mL. Conventional HDL nomenclatures are based on physical properties such as density [9] or composition [10]. HDL is constituted by a large number of heterogeneous particles differing in size, charge, shape, lipid composition (glycerophospholipids, triglycerides, sphingolipids, cholesterol, and cholesteryl esters), and physiological functions [11].Proteins constitute approximately 50% of HDL protein mass with apoA-I representing approximately 65–70%, with another 12% to 15% being apoA-II. Recent proteomic studies have identified more than 60 different proteins on HDL adding to the biological diversity of this class of lipoproteins [12]. HDL is also highly heterogeneous with regard to its lipidome [7]. Thus, the term HDL applies to a large and heterogeneous group of small (5–70 nm) particles with diverse lipids and proteins [12, 13] that may differ in function [11]. Static mass-based measurement of HDL-C may be an imperfect metric of HDL functionality, particularly in the setting of therapeutic interventions. Proposed definitions of HDL rely on diverse analytical techniques; a unified definition is emerging [7], although consensus is not yet reached [8]. Rosenson et al. [7] proposed a nomenclature based on particle size and density. Other argue that this classification is incomplete and further characterization should be made for HDL of lower density, larger size, and protein components (especially apo E) which may have better discriminant power in the clinical setting [8]. Despite this, the relevance of HDL subfractions to CVD remains ambiguous and lacks standardization and validation [11, 14]. There is a need to refine definitions of HDL to encompass the functional qualities of HDL. It is hoped that the adoption of a uniform nomenclature system for HDL subfractions that integrates several methods will enhance our ability to assess the clinical effects of different compounds that modulate HDL metabolism, function, and structure, and in turn, allow improved cardiovascular risk prediction.

## 3. Epidemiology of HDL and CVD

Epidemiological studies have shown an inverse relationship between HDL-C levels and CVD risk [15–17]. This negative association is strong, graded, and coherent across population studied and has led to the development of the “HDL-C hypothesis,” which proposes that pharmacological intervention to raise HDL-C will reduce cardiovascular risk. However, there is controversial data suggesting that certain patients at high cardiovascular risk have “dysfunctional” HDL despite normal HDL-C levels [16]. Importantly, the association of HDL-C with CVD risk is further confounded by the inverse association of HDL-C with apo B and LDL particles concentrations. Many other variables such as triglycerides, insulin resistance, obesity, and C-reactive protein may bias this association. Despite the data generated from the Emerging Risk Factor Collaboration [17], it may be impossible to take all these factors into account. Indeed in the Multi-Ethnic Study of Atherosclerosis (MESA) in 5,598 volunteer without baseline CHD, Mackey et al. showed that the association of HDL-C and carotid disease was considerably attenuated when taking into account atherogenic lipoproteins [18].

## 4. Clinical Trials

Fibric acid derivatives (fibrates) are peroxisome proliferator-activated receptor alpha (PPAR*α*) agonists and their major role is to increase lipoprotein lipase; they raise HDL-C modestly, in the range of 10–15%. While early reports of fibrates have shown encouraging results in terms of cardiovascular outcomes,these were performed before the statin era and the results are difficult to interpret in light of the effects of fibrates on LDL-C. Recent studies using fenofibrate in diabetic patients failed to show an effect on cardiovascular outcomes when LDL-C was controlled with statin therapy [19]. A recent meta-analysis of fibrate trials reveals that while fibrates may decrease the risk of nonfatal cardiovascular events and on microvascular disease, their overall effect on cardiovascular mortality is neutral [20]. Statins are widely acknowledged as first-line drugs for the treatment of dyslipidemias and CVD prevention; however, statins may not address residual risk [21]. Significant residual cardiovascular risk remains even after intensive statin therapy and lowering LDL-C to <70 mg/dL [22]. Meta-analysis studies show that reduction of LDL cholesterol by 1 mmol/L reduces risk by about approximately 20–25% and that intensive LDL-C lowering with statins lowers risk by up to 33% [3]. Despite this, HDL-C levels continue to predict risk in statin-treated patients [23, 24]. This has been challenged in clinical trials where LDL-C has reached a very low level [25, 26]. Attempts to decrease cardiovascular risk in statin-treated patients with the CETP inhibitor torcetrapib have failed, despite an increase in HDL-C by 72% [25]. In the ILLUMINATE study [25] adverse events caused by torcetrapib were likely to represent off-target effects of the drug but raised question about the value of raising HDL-C. The dal-OUTCOMES trial using dalcetrapib raised HDL-C by 31 to 40% [27] but had no effects on cardiovascular events. Niacin, used at pharmacological doses (up to 2 gm/day), led to a neutrality of results in terms of cardiovascular outcomes in the AIM-HIGM [4]. The recent failure of the large (>25,000 subjects) HPS2-THRIVE trial with niacin [28] raised questions about the benefits of this therapeutic strategy to raise HDL. The clinical equipoise remains. The failure of torcetrapib and dalcetrapib may be explained by off-target adverse effects and weak CETP inhibition, respectively. AIM-HIGH has been criticized for having a relatively small sample size [29].

## 5. HDL Genetics

Genes that modulate HDL-C have been characterized by conventional family studies and by genome-wide association studies. Yet not all genetic forms of very low HDL-C and apoA-I are necessarily associated with increased risk of CVD. Tangier disease, in which HDL-C and apoA-I concentrations are virtually undetectable, is not associated with a marked increase of CVD that might be expected from such a dramatic phenotype [1]. Recent Mendelian randomization studies have shown that patients with mutations in the ABCA1 gene (Tangier disease) are not at increased cardiovascular risk in the Copenhagen Heart Study [30] and many mutations within apo AI that cause a very low HDL-C and apo AI are not associated with premature CVD. Thus, genes causing HDL-C deficiency are not necessarily associated with increased cardiovascular risk. Voight et al. recently reported that genes that are associated with an increase in HDL-C (endothelial lipase and CETP) are not associated with decreased cardiovascular risk [31]. Thus, Mendelian randomization experiments have cast into doubt the link of causality between HDL-C and risk of CVD.

## 6. HDL Functions

### 6.1. Reverse Cholesterol Transport (RCT)

**Figure 1:**
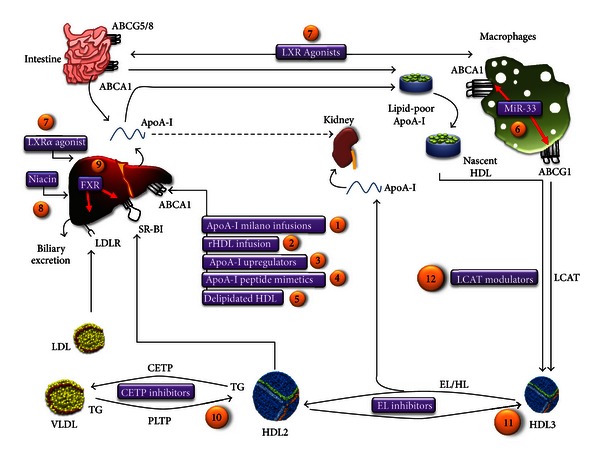
Schematic diagram of HDL metabolic pathways and current drugs under development. Numbers in circles refer to Table 2. Pathway influencing HDL cholesterol metabolism, flux, and potential targets of therapeutic interventions. Both liver and intestine synthesize apolipoprotein A-I (ApoA-I) secreted as lipid-poor particles. These particles are lipidated with phospholipids and cholesterol via the hepatocyte ATP-binding cassette A1 (ABCA1) transporter to form nascent HDL. In peripheral tissues these HDL particles obtain free cholesterol via the macrophage ABCA1 and ABCG1 transporters, which are regulated by LXRs and miR-33. Free cholesterol transferred via ABCA1 and ABCG1 onto HDL is esterified by lecithin: cholesterol acyltransferase (LCAT) to form cholesteryl esters (CE). Mature HDL thus formed exchange CE trough cholesteryl ester transfer protein (CETP) onto apoB-containing lipoproteins, (VLDL and LDL) with subsequent uptake in the liver via the low-density lipoprotein receptor (LDLR). PLTP mediates transfer of phospholipid from triglyceride from VLDL into HDL, which promote HDL remodeling. The resulting HDL3 particles can be either taken up by the liver via SB-B1 or modified by hepatic lipase (HL) and endothelial lipase (EL), which hydrolyze HDL phospholipids and triglycerides. Metabolism by EL releases lipid-poor apoA-I, which can be catabolized in kidney. Targets of HDL-directed therapeutic interventions are indicated by red arrow.

**Figure 2:**
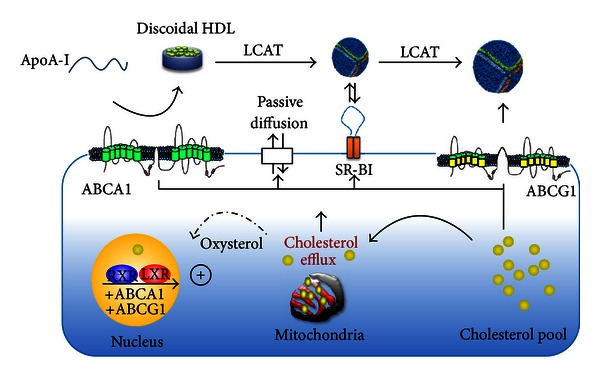
HDL biogenesis. Mitochondrial cholesterol transport is rate limiting in the (sterol 27-hydroxylase-) dependent generation of oxysterol ligands for LXR (liver X receptor) transcription factors that regulate the expression of genes encoding proteins in the cholesterol efflux pathway, such as ABC transporters (ATP-binding cassette transporters) ABCA1, and ABCG1. These transporters transfer cholesterol and/or phospholipids across the plasma membrane to (apo) lipoprotein acceptors, generating nascent HDLs (high-density lipoproteins), which can safely transport excess cholesterol through the bloodstream to the liver for excretion in bile. Ligand activation of nuclear LXRs (liver X receptors) (LXR*α*/*β*) is pivotal in cellular response to elevated sterol content, triggering cholesterol efflux mechanisms: both synthetic and oxysterol LXR agonists potently upregulate ABCA1 and ABCG1 gene expression. Consequently, elimination of excess cholesterol can be achieved, *in vivo* and *in vitro*, by cellular cholesterol efflux, orchestrated by ABCA1, ABCG1, ABCG4, and passive diffusion along a concentration gradient, and also “acceptor” (apo) lipoproteins, such as apoA-I, and HDLs (high-density lipoproteins). Notably, LXRs form heterodimers with RXRs (retinoic acid receptors) and bound to the nuclear receptor.

The concept of RCT was first proposed by Glomset [32] in 1968. This concept represents the most widely accepted mechanism underlying the HDL-C hypothesis. Free cholesterol (FC) is toxic to cells. In the endoplasmic reticulum, cholesterol may initiate the unfolded protein response, leading to cell apoptosis [33]. Cellular cholesterol homeostasis is exquisitely maintained and the control of *de novo* synthesis and LDL-receptor-mediated endocytosis remains critical for this process. Cellular cholesterol can also be modulated by efflux. This is mediated via different pathways, which are transporter-independent or depend on the receptor/transporter scavenger receptor class B type I (SR-BI), ATP-binding cassette receptors ABCA1 and ABCG1 (Table 1) (Figures 1 and 2). A measure of HDL-mediated cellular cholesterol efflux from macrophages may provide a novel way to the assessment of CVD risk. It has been shown that cholesterol efflux from macrophages is strongly associated with atherosclerosis and provides better CVD prediction than plasma HDL-C levels [34]. The presence of specific subparticles, especially pre-*β* HDL, may provide a biomarker for CVD prevention and represent a potential therapeutic target. Thus, identifying biomarkers of HDL quality and function may provide a better discriminant for CVD than HDL-C mass. In turn, these biomarkers can be used to gauge the effects of therapies and be correlated with outcomes. Mechanisms of cholesterol efflux are discussed below.

### 6.2. Aqueous Diffusion

Aqueous diffusion involves a simple diffusion process in which FC molecules desorb from the plasma membrane, move through the extracellular aqueous space, and are then rapidly incorporated into the surface of HDL particles by gradient diffusion (Figure 2). Our data indicates that approximately 1-2% of cellular cholesterol efflux occurs via this pathway [35]. Aqueous diffusion is the rate limiting step in desorption of FC from the cell membrane into surrounding aqueous phase. This rate is increased by higher phospholipid unsaturation and decreased by higher membrane sphingomyelin content [36] and dependent on the structure and particle size of acceptor particle. Rothblat and Phillips [37] reported recently that larger HDL2 and smaller HDL3 particles are equally effective as acceptors in this efflux mechanism, suggesting that in this pathway the efflux process is not affected by alteration in HDL particle size. The importance of this pathway in maintaining cellular cholesterol homeostasis and preventing atherosclerosis is not clear. A pathway analysis on this protocol using previously published techniques indicated that in mouse peritoneal macrophages 34% of the total efflux was mediated by ABCA1, 20% by SRB1, and 46% by ABCG1 and/or, aqueous diffusion, or undiscovered pathways [38].

### 6.3. ABCA1

ABCA1 mediates the rate limiting step in HDL biogenesis, namely, the transfer of cellular cholesterol and phospholipids onto lipid-poor apolipoprotein acceptor to form nascent HDL particles. In turn, these particles undergo further lipidation via ABCG1 [39]. ABCA1 mediated cellular cholesterol movement is unidirectional, resulting in net efflux (Figure 2). In this pathway lipid-free apoA-I is the primary acceptor of cholesterol. HDL lipidation of apoA-I by ABCA1 is a complex process and may involve more than a direct interaction with this transporter. The nature of the molecular interactions between apoA-I and ABCA1 continues to generate controversy, and models suggesting a direct protein-protein interaction or indirect association have been proposed. Experimental data supports the concept that apoA-I directly binds to ABCA1 in the process of HDL formation, as shown via cross linking experiments within a distance of ≤3 Å [40]. Indeed, cholesterol export function of ABCA1 occurs by a cascade of events involving direct binding of apoA-I to ABCA1, activation of signaling pathways, and solubilization of cholesterol and phospholipids rich membrane domains formed by ABCA1 [41]. This interaction is localized on the cell surface [42] and results in the formation of nascent HDL particles that exhibit size (7–12 nm) and lipid composition heterogeneity, confirming that apoA-I exhibits considerable flexibility and conformational adaptability to its lipid constituents [42, 43]. ApoA-I stabilizes ABCA1 and prevents calpain-mediated degradation [44]. Thus, apoA-I increases the amount of ABCA1 on the plasma membrane and promotes cholesterol transport from intracellular compartments to the plasma membrane and ABCA1 also facilitates mobilization of cholesterol from late endocytic compartment to the cell plasma membrane [45]; the contribution to net efflux via this mechanism is controversial [46]. Many tissues express ABCA1; the liver and intestine are the major organs contributing to plasma HDL-C levels [47, 48]. Other tissues, such as adipocytes have been shown to be important site for HDL formation and lipidation [49]. Recently, an intronic microRNA, located within the SREBF2 gene, suppresses expression of the cholesterol transporter ABCA1 and lowers HDL levels in mouse and human cells [50]. Conversely, mechanisms that inhibit miR-33 increase ABCA1 and circulating HDL levels, suggesting that antagonism of miR-33 may be atheroprotective. Recently, treatment with an antisense oligonucleotide in mice was shown to increase macrophage reverse transport and decrease atherosclerosis [51]. In nonhuman primates, treatment with a miR-33 antisense oligonucleotide was shown to increase HDL-C up to 30% [52]. Strategies to silence miRNA may be a therapeutic in man. The ABCA1 pathway represents an important therapeutic target for the prevention and treatment of CVD (Figure 1, part 6).

### 6.4. ABCG1

ABCG1 is a half transporter that homodimerizes to form a full transporter. Unlike ABCA1, lipid-free or lipid-poor apoA-I does not bind to ABCG1 and does not mediate cholesterol efflux. ABCG1-mediated cholesterol efflux requires larger HDL particles and this efflux is unidirectional against a concentration gradient [38] (Figure 2). In macrophages, ABCG1 is an LXR target which was subsequently shown to promote mobilization of intracellular cholesterol [53] to spherical HDL particles; HDL3 particles being the most efficient [54]. In addition to LXR activation, ABCG1 expression can also be induced by agonist of peroxisome proliferator-activated receptor gamma (PPAR*γ*) associated with increased LXR [55]. Regulation of these pathways is complex and recent data shows that miR-33 increases ABCG1 expression *in vivo* [56]. In mouse macrophages, miR-33 targets ABCG1 thereby reducing cholesterol efflux to nascent HDL [50, 56]. These data demonstrate that miR-33 deficiency or inhibition raises HDL-C, increases cholesterol efflux from macrophages via ABCA1 and ABCG1, and prevents the progression of atherosclerosis. However, many genes are altered in miR-33-deficient mice, and more experimental data is required to establish miR-33 as a therapeutic modality in humans. Gelissen et al. have characterized two novel isoforms of ABCG1 in human vascular endothelial cells that induce different levels of cholesterol efflux [57]. New insights into coordinated participation of ABCA1 in concert with ABCA1 in promoting cellular cholesterol export to lipid-free/poor apoA-I have evolved; ABCA1 initially donates phospholipids and cholesterol to lipid-poor apoA-I; ABCG1 mediates further cellular efflux onto more mature HDL particles [58]. These transporters thus work synergistically to remove excess cholesterol from cells (particularly macrophages). Murine models of atherosclerosis, using ABCG1-apoE double knock out, showed markedly defective cholesterol efflux and increased macrophage apoptosis [59]. Conversely, overexpression of ABCG1 increased the rate of constant efflux of cholesterol and protected murine tissues from lipid accumulation [60, 61]. The potential importance of ABCG1 in RCT has not been fully established.

### 6.5. SR-BI

SR-BI is critical in facilitating the delivery of cholesterol from macrophages to the liver. The SR-BI pathway acts as a receptor for large HDL particles (HDL2) and mediates the selective uptake of cholesteryl esters, without incorporation of the holoparticle [36, 54] (Figure 2). SR-B1 also mediates cellular cholesterol efflux onto HDL particles. HDL phospholipids absorb cholesterol that diffuses from the plasma membrane by the interaction between HDL and SR-BI [36, 37]. The relationship of hepatic SR-BI expression to HDL-C levels and atherosclerosis is ambiguous in light of human epidemiologic data. Several studies established a role of SR-BI expressed or inhibited in liver and in bone marrow-derived cells in the protection against atherosclerosis [62, 63]. Carriers of the first reported mutation of SR-BI in humans (*SRB1* P297S) have increased HDL-C levels but reduced capacity for cholesterol efflux from macrophages without increased severity of atherosclerosis [64]. Selective disruption of SR-BI in bone marrow-derived cells, including macrophages, leads to accelerated atherosclerosis indicating a dual role in atherosclerosis lesion development [62]. Mouse SR-BI^−/−^ models exhibit increased HDL-C concentrations, decreased markers of RCT, and increased atherosclerosis [65]. *In vivo* data demonstrated that SR-BI does not promote macrophage RCT as seen with ABCA1 and ABCG1 [66]. The hepatic SR-BI may have an indirect role in atherosclerosis by modulating changes in the composition and structure of HDL particles rather than changes in the HDL pool [65, 67, 68]. A novel blocker of SR-B1, ITX5061 (a molecule initially characterized as p38 mitogen-activated protein kinase (MPK)), increases HDL-C in mice and decreases the formation of early atherosclerosis lesions. In human, SR-BI blockade by ITX5061 increases HDL-C levels without adverse effects on VLDL/LDL cholesterol, although this effect seems to be transient [69]. This data supports the concept that ABCA1, ABCG1, and SR-B1 act cooperatively in the peripheral biogenesis of nascent HDL particles, maturation of HDL, and selective CE uptake by the liver, respectively, completing the RCT in the plasma compartment.

## 7. Functions of HDL

### 7.1. Antioxidant

**Figure 3:**
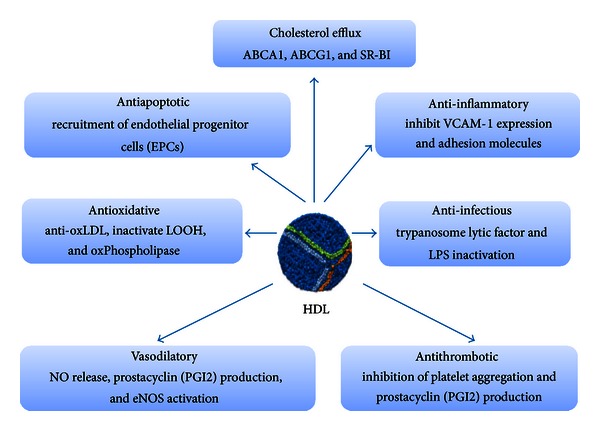
Summary of pleiotropic effects of HDL. In addition to their ability to reverse transport cholesterol from peripheral tissues back to the liver, HDLs display pleiotropic effects including antioxidant, antiapoptotic, anti-inflammatory, anti-infectious, and antithrombotic properties that account for their protective action on endothelial cells. Vasodilatation via production of nitric oxide (NO) is also a hallmark of HDL action on those cells.

Oxidative modification of lipids, especially fatty acyl residues within phospholipids, leads to free radical formation and cell damage. Products of lipid peroxidation present in oxLDL can induce proinflammatory phenotypes in arterial wall cells, which contribute to endothelial dysfunction and apoptotic cell death, key steps in the initiation and progression of atherosclerosis lesions [70]. HDL prevents LDL from free radical-induced oxidative damage through several mechanisms [71, 72] (Figure 3). Several antioxidant enzymes that are involved in prevention of lipid oxidation or degradation of lipid hydroperoxides, such as LCAT, platelet-activating factor acetylhydrolase, reduced glutathione selenoperoxidase, and paraoxonase 1 (PON 1), are present on HDL. This antioxidative activity of HDL appears to be defective in dyslipidemic patients at high CVD risk [73]. Measuring the antioxidant properties of HDL is cumbersome; novel approaches use indirect measurements of phospholipid oxidation. The HDL oxidant index is an assay based on the chemoluminescence emitted by dichlorofluorescin that quantifies the antioxidation activity of HDL. This assay has been used as a biomarker of HDL functionality in experimental models and in therapeutic interventions in man [74]. Several biomarkers of oxidation potential have been put forth, by measuring the formation of biologically active oxidized phospholipids, by the determination of endothelial superoxide production and NADPH oxidase, or by the measurement of paraoxonase activity [73].

## 8. Vascular Endothelial Function of HDL

### 8.1. Anti-Inflammatory

Atherosclerosis is an inflammatory disease. HDL prevents vascular inflammation (Figure 3) through various pathways, in part via modulation of nuclear transcription factor *κ*b- (NF-*κ*b-) activated cell adhesion molecules [71] and via macrophage activation of JAK2/STAT3 interaction with apo AI/ABCA1 complex [75]. *In vitro* studies have demonstrated that HDL inhibits the expression of endothelial adhesion molecules, such as vascular cell adhesion molecule-1 (VCAM-1) and E-selectin [71]. Expression of endothelial adhesion molecules by HDL has also been demonstrated in the aortic endothelium of rabbits *in vivo* [76]. Recently, elegant experiments by Besler et al. have shown that modulation of vascular inflammation by dysfunctional HDL is mediated by upstream regulation of nuclear factor *κ*-B (nf-*κ*B) [71, 77]. Another anti-inflammatory effect of HDL involves the transport of sphingosine-1-phosphate (S1P) a lipid mediator that has anti-inflammatory actions at low concentrations [78]. In human aortic endothelium and smooth muscle, HDL prevents the expression of IL-8 and monocyte chemoattractant protein (MCP-1) under inflammatory conditions [70], showing that normal HDL is capable of preventing LDL oxidation and the inflammatory response induced by LDL. During inflammation, HDL cargo becomes oxidatively and enzymatically modified, and HDL loses its protective capacity [70]. In the setting of an acute coronary syndrome (ACS), HDL promotes LDL-induced endothelial MCP-1 expression and monocyte adhesion [71]. Moreover, HDL from these patients reflects a shift to an inflammatory profile which, in turn, might alter the protective effects of HDL on the atherosclerotic plaque [73, 79]. To study the function and the effects of HDL in setting of inflammation, a cell free assay for measuring lipid hyperoxides in plasma was developed to determine the inflammatory properties of HDL [74].

### 8.2. Vascular Endothelial eNOS

HDL-derived cholesterol has been shown to promote endothelial generation of nitric oxide (NO) *in vitro* and to improve endothelial function and arterial vasoreactivity *in vivo* (Figure 3). Activation of NO production involves HDL binding to SR-BI, which activates the phosphatidylinositol-3-kinase (PI3 K)/Akt signalling pathway and the phosphorylation of endothelial nitric oxide synthase (eNOS) [6]. In previous studies it has been shown that eNOS activation in response to HDL is mediated via Akt-dependent eNOS phosphorylation at Ser1177 [80]. Notably, Besler et al. showed that HDL from patient with CAD, in contrast to HDL from healthy subjects, activates endothelial PKC*β*II, leading to inhibition of Akt-dependent eNOS activating phosphorylation at Ser1177 and increased phosphorylation of eNOS at Thr495, which inhibits eNOS activity [6]. Experimental studies have consistently demonstrated the capacity of HDL to modify eNOS expression as well as activity to stimulate endothelial NO production *in vitro* and *in vivo* [6, 71, 73]. Several mechanisms have been shown that modulate eNOS and NO production by HDL [71]. The HDL-associated PON-1 has been shown to be an important determinant of the capacity of HDL to stimulate endothelial NO production and to exert NO-dependent endothelial-atheroprotective effects [6]. As observed in patients with CAD, inhibition of HDL-associated PON1 led to the increase of malonaldehyde- (MAD-) lysine adducts in HDL that subsequently activate protein kinase C beta II (PKC*β*II) via the endothelial LOX-1 receptor [6, 71].

### 8.3. Antiapoptotic

Cell apoptosis in response to endothelial injury is an important feature in atherosclerosis and is, in part, stimulated in part by oxLDL (Figure 2). It has been shown that HDL is able to protect oxLDL-induced apoptosis by blocking intracellular signaling involved in apoptosis [81]. HDL promotes vascular cell migration, proliferation, survival (antiapoptotic), and recruitment of endothelial progenitor cells (EPCs) to sites of vascular injury [17, 82]. Recently, Riwanto et al. demonstrated that HDL from healthy subjects induced expression of the antiapoptotic Bcl-2 protein Bcl-xL from endothelial cells and reduced endothelial cell apoptosis. This effect, however, was lost when HDL from subjects with coronary artery disease was used [77].

### 8.4. Antithrombotic

HDL may exert antithrombotic activity via inhibition of tissue factor expression, factor X activation, and plasminogen activator inhibitor secretion [83]. HDL blocks the formation of the platelet activator thromboxane A2, and platelet activation factor synthase (Figure 3), thus reducing platelet aggregation [6]. Surrogate measures of HDL antiplatelet and antithrombotic functionality remain underdeveloped, in large part because of the absence of consensus regarding the clinically relevant atheroprotective pathways. Thus, there is strong biological plausibility for targeting HDL-C for the prevention and treatment of CVD. Controversy remains, however, in light of the apparent controversies summarized in Table 2.

### 8.5. Anti-Infectious (Antitrypanosome)

HDL displays anti-infectious activity in the binding and clearance of circulating bacterial lipopolysaccharide (LPS) to the bile and thereby inhibits endotoxins-induced cellular activation, resulting in potent anti-infectious activity (Figure 3). Mechanism of LPS inactivation by HDL is mediated by direct interaction with apo A-I and involves reduced CD14 expression on monocytes as a key step [84]. Apo A-I overexpression in mice diminishes significantly LPS-induced systemic inflammation and multiple organ damage [84]. HDL toxin-neutralizing activity is largely attributed to apo A-I and has been furthermore shown to be effective against enterohemolysin [85] and lipoteichoic acid [84]. More recently, a study demonstrates that apoA-I reduces LPS-induced inflammatory responses, both *in vitro* and *in vivo*, and inhibits the development of atherosclerosis [86]. Furthermore, in cattle, the *Trypanosoma brucei brucei* causes a devastating disease, nagana. In man, HDL prevents trypanosomes from infecting cells by forming a complex with haptoglobin and causing increased permeability to water in *T. brucei brucei*, causing lysis [87].

## 9. Novel Therapies under Development to Raise HDL-C Levels

Table 3 and Figure 1 summarize selected strategies to increase HDL/apoA-I and describe potential compound under development.

## 10. CETP Inhibitors (Figure 1, Part 10)

Torcetrapib was the first CETP inhibitor to be used in a large-scale clinical trial. The ILLUMINATE trial failed to show benefit from the drug and the clinical trial was brought to a sudden and unexpected halt because of increased mortality in subjects on torcetrapib. This effect is thought to represent off-targets effects, predominantly on blood pressure control of torcetrapib [25]. A second, weaker CETP inhibitor, dalcetrapib increased HDL-C by 31–40%, without changing LDL-C. The study showed no benefit on cardiovascular outcomes [27]. Two new CETP inhibitors (anacetrapib and evacetrapib) are in phase III clinical trials.

Anacetrapib (MK-0859) (Table 3) raises HDL without affecting blood pressure [88]. The Determining Efficacy and Tolerability (DEFINE) trial randomized 1623 patients with CAD whose statin treatment has achieved LDL < 100 mg/dL to 100 mg of anacetrapib or placebo [89, 90]. Anacetrapib therapy resulted in 138% increase in HDL-C, a 40% reduction in LDL-C, and a 36% decrease in Lp[a] [90]. The off-target effects seen with torcetrapib were not identified [91]. The large REVEAL HPS-3/TIMI-55 trial will test the hypothesis that lipid modification with anacetrapib 100 mg daily reduces the risk of coronary death, myocardial infarction, or coronary revascularization in 30,000 patients with CVD or diabetes on optimal statin treatment with atorvastatin. (ClinicalTrials.gov NCT01252953) The estimated study completion date is 2017 [92].

Evacetrapib is a benzazepine compound (LY248595) and a potent and selective inhibitor of CETP both *in vitro* and *in vivo* (Table 3). Clinical trials with evacetrapib showed substantially increased HDL-C (54–129%) and decreased LDL-C (14–36%) across a dose range of evacetrapib in 398 dyslipidemic patients [93]. In this trial evacetrapib has shown no demonstrable effects on blood pressure or adrenal synthesis of aldosterone or cortisol in preclinical studies. The effects of evacetrapib on cardiovascular outcomes are being examined in the Assessment of Clinical Effects of Cholesteryl Ester Transfer Protein Inhibition with Evacetrapib in Patients at a High-Risk for Vascular Outcomes (ACCELERATE study), enrolling 11,000 patients after ACS [94].

## 11. Niacin (Figure 1, Part 8)

Niacin (nicotinic acid) is the oldest of the lipid-lowering drugs [95]. The mechanisms by which niacin raises HDL-C remain poorly understood, despite the identification of specific cell receptors (GP109, HM74, and HM74A). Niacin lipid efficacy is independent of both the niacin receptor GPR109A and free fatty acid suppression [91]. Niacin effects involve reducing the synthesis of VLDL in the liver through diaglycerol acyl transferase-2 (DGAT-2) as well as affecting peripheral lipolysis [96]. Niacin was also shown to have pleiotropic effects, such as improving endothelial-protective functions of HDL in patient with type 2 diabetics with low HDL-C levels [73]. In the early lipid-lowering drug therapy the Coronary Drug Prevention project [97], niacin alone was shown to reduce myocardial infarction and stroke in a randomized 3906 patients with previous myocardial infarction. Later, a meta-analysis study of 11 randomized controlled trials, using niacin (none in monotherapy), was shown to significantly reduce major coronary events by 25%, stroke by 26%, and all cardiovascular events by 27% [98]. Two new, large-scale clinical trials examined the role of niacin as an add-on to statin therapy for the secondary prevention of CVD. The Atherothrombosis Intervention in Metabolic Syndrome with Low HDL/High Triglycerides: Impact on Global Health Outcomes (AIM-HIGH) trial randomized 3,414 statin-treated CAD patients with low HDL-C to niacin versus placebo [4]. This trial was stopped prematurely because the lack of benefit on cardiovascular outcomes, despite a 10% increase in HDL-C [99]. The HPS-2/THRIVE (Heart Protection Study-2-Treatment of HDL to Reduce the Incidence of Vascular Events), a study of 25,000 patients at high risk for CVD events [5, 100] examined the effects of niacin and laropiprant on cardiovascular outcomes. After a mean followup of 3 years the combination of niacin/laropiprant therapy added to statin therapy did not reduce CVD risk events compared to statin therapy alone. Further, nonfatal attacks, strokes, or revascularizations were increased in participant who received niacin plus laropiprant. Niacin has many side effects, including hepatotoxicity, hyperglycemia, and flushing [101]. Non-flushing niacin derivatives have been synthesized such as prostaglandin D2 DP1 receptor antagonist-laropiprant. In the phase 3 trials niacin-laropiprant 2 g shows reductions in TG by 23%, 18% in LDL-C, and a 20% increase in HDL-C [102]. Another approach is, ARI-3037MO (Arisaph Pharmaceuticals; Boston, MA, USA) presently in phase I trials [103]. The success of this approach has been put into doubt following AIN-HIGH and HPS2 results (Table 3).

## 12. Directly Augmenting Apo A-I and ApoA-I/Phospholipid Complexes (Figure 1, Parts 1, 2)

This concept was developed to directly increase HDL, by infusing reconstituted HDL (rHDL) or recombinant HDL particles into the circulation rather than increasing HDL indirectly by modulating HDL metabolism (Table 3). One approach uses recombinant apoA-I_Milano_. Individuals with apoA-I_Milano_ mutation have low HDL-C levels (10 to 30 mg/dL), and no apparent increased CVD risk [104]. Early studies indicated that recombinant apoA-I_Milano_, when delivered by intravenous infusion, promotes regression of atherosclerosis lesion to a greater extent than wild type apoA-I as measured by intravascular ultrasound within 5 weeks of treatment [105]. Procedural difficulties complicated the development of ETC-216 (clinical denomination of apoA-I_Milano_) [106] and no further clinical trials with this formulation have been reported [107]. More recently, it has shown that recombinant HDL apoA-I_Milano_ exerts greater anti-inflammatory and plaque stabilizing properties rather than antisclerotic properties [108]. Another rHDL compound, CSL-111 consists of apoA-I purified from human plasma and complexed with phosphatidylcholine derived from soybean phosphatidylcholine. The first trial of CSL-111 examined the effect of rHDL in the Atherosclerosis Safety and Efficacy (ERASE) trial conducted in 183 patients with ACS [106]. Four weekly infusion of CSL-111 of 4 h each to 111 individuals randomized to the 40 mg/kg proved to be well tolerated and failed to meet its primary end-point. The high dose regimen (80 mg/kg) was discontinued because of abnormal liver transaminase elevations. However, there was no significant change in atheroma volume, as measured by IVUS, compared with the placebo group. Since ERASE, there is one other randomized study investigating the effect of CSL-111 on surrogate cardiovascular marker in patients with ACS [109]. In this trial 29 patients were randomized to single infusion of CSL-111 (80 mg/kg over 4 h) or albumin. An increase of HDL (64%) and a reduction of LDL (23%) notwithstanding human rHDL did not improve vascular function in patients with ACS and there was no significant difference between the two arms suggesting no benefit with a single infusion. CSL-111 and also ECT-216 are currently in phase II trials. A reformulated version of CSL-111, called CSL-112, has been reported in preclinical studies. A phase I safety study has been initiated [110].

## 13. Delipidated HDL Infusions (Figure 1, Part 5)

Delipidated HDL infusion is a novel approach to raise HDL by intravenous infusion of autologous delipidated HDL [111] (Table 3). Preclinical evaluation of selective delipidated HDL in dyslipidemic monkeys achieved a significant 6.9% reduction in aortic atheroma volume assessed by IVUS [112]. The process involves the selective removal of apoA-I HDL particles, and the delipidation reinfusion of the cholesterol-depleted functional pre-*β* HDL. In a human trial, 28 patients with ACS received 5 weekly infusions delipidated HDL (*n* = 14) or placebo (*n* = 14). Study results established that selectively increasing pre*β*-HDL was associated with decreased total atheroma volume by 5.2% from base line [111]. However, it is not clear whether acute regression of atherosclerosis burden will be associated with decreased clinical cardiovascular events. Autologous delipidated HDL infusions did not induce liver toxicity or hypersensitivity reactions. In the study HDL apheresis resulted in hypotension in a third of the participants undergoing the treatment. A delipidation system for human use is now evaluable from lipid Sciences Plasma Delipidation System-2 (PDS-2), which converts *α*HDL to pre*β*-like HDL by selectively removing cholesterol from HDL in samples of plasma collected from patients by apheresis.

## 14. HDL Mimetics

### 14.1. ApoA-I Mimetic Peptides Drugs (Figure 1, Part 4)

ApoA-I mimetics are short synthetic peptides that mimic the amphipathic *α*-helix of apoA-I.The first apoA-I mimetic peptide consisted of 18 amino acids (compound 18A) [113]. The sequence of 18A (D-W-_L_-K-A-F-Y-_D_-K-V-A-E-K-_L_-K-E-A-F) does not share sequence homology with apoA-I because of the presence of two phenylalanine (F) residues on the hydrophobic face at positions 6 and 18; it is also referred to as 2F. While this sequence has the capacity to assume *α*-helical configuration in secondary structure and mimics many of the lipid binding properties of apoA-I [114], it failed to decrease diet induced atherosclerosis in mice [115]. Consequently, from 18A structure, additional improved peptides were generated through increasing number of phenylalanine residues on the hydrophobic face (referred to as 2F, 3F, 4F, 5F, 6F, and 7F) [115]. Among the three first peptides, only 4F was highly effective in preventing LDL-induced MCP-1 production by cultured human artery wall cells [115]. Shah and Chyu [116] found that D-4F reduced vein graft atherosclerosis in apoE null mice fed a western diet. Weihrauch et al. [117] gave intraperitoneal injection in a mouse model of systemic sclerosis, deduced that D-4F decreases myocardial inflammation index, improves vascular function, and restored angiogenic potential [118].Optimal physical-chemical properties and biologic activity were found with compound 4F [119]. The apoA-I mimetic peptide 4F showed great promise in a number of animal models and in early human trials [120] leading to a phase I/II study in humans with high risk CVD [118]. In this study, the 4F peptide synthesized from all _L_-amino acids for L-4F was delivered at low doses (0.042–1.43 mg/kg) by intravenous or subcutaneous administration [121]. Very high plasma peptide levels were achieved, but there was no improvement in HDL anti-inflammatory function [121]. On the other hand, data showed that L-4F restores vascular endothelial function in murine models of hypercholesterolemia [122]. In another clinical trial the 4F peptide synthesized from all _D_-amino acids for D-4F was administered orally at higher doses (0.43–7.14 mg/kg). Interestingly, despite very low plasma peptide levels, it has shown significantly improved HDL inflammatory index [118]. In humans with significant cardiovascular risk, a single dose of D-4F was found to improve the inflammatory index of HDL with modest oral bioavailability [119]. In contrast to L-4F, D-4F is poorly degraded in mammals, which led to its prolonged tissue retention time, particularly in liver and kidneys [114]. Despite these differences, the effects of D-4F and L-4F on biomarker and lesion area were similar when administered by subcutaneous injection in cholesterol fed rabbits [119]. In addition, oral D-4F administration in mice can influence several steps in the RCT pathway by increasing plasma LCAT activity, but the effect is likely due to HDL remodelling rather than activation of the enzyme [123]. Recent studies in mice revealed that the site controlling 4F peptide efficiency might be the small intestine even when it is delivered subcutaneously [124, 125]. Interestingly, the dose administered, not the plasma level, was the major determinant of efficiency of the 4F peptide [124]. At high doses, D-4F displayed detergent-like properties and could extract cholesterol from cells independently of ABCA1 [114, 126]. Unfortunately, D-4F may exhibit cytotoxicity through ABCA-1 independent lipid efflux. Furthermore, these mimetics have end-blocking groups (Ac- and NH_2_) which can only be added by chemical synthesis. These blocking groups for 4F and for many other apoA-I mimetics stabilize the class A amphipathic helix and dramatically increase efficiency [127].

Recently, peptide 6F—that do not require added chemical groups for efficacy—has been produced in genetically engineered tomatoes. Compound 6F produced by this mean showed anti-inflammatory properties when fed orally to mice, providing a novel approach to orally administered apoA-I mimetic compounds [128]. Of the apoA-I mimetics synthesized to date, both 5F and 6F were able to efficiently inhibit LDL-induced MCP-1 production by cultured human artery wall cells, however, 7F did not [129]. 5A peptide was also effective as rHDL in reducing the expression of VCAM-1 and ICAM-1, and similarly to HDL exerted its effects through ABCA1 *in vivo* and *in vitro* [130] (Table 3).

### 14.2. ATI-5261 Synthetic Peptide

Native apoA-I is a 243 amino acid protein that contain multiple *α*-helical repeated in tandem and separated by proline residues. Most of *α*-helices are 22 amino acids long with a unique secondary structure defined as class A amphipathic *α*-helix. In this helix, negatively charged amino acid residues are clustered at the center of the polar face and positively charged residues are at the interface between the hydrophilic and hydrophobic faces; *α*-helices linked via proline are required to support potent biological activity [43]. Studies using the 22-mer amphipathic *α*-helix of apoA-I have not been able to stimulate ABCA1-dependent cholesterol efflux [131]. Short sequences taken directly from native proteins often cannot typically replicate the biological activity of the parent peptide [132], possibly due to peptide aggregation via nonspecific hydrophobic interactions and decreased solubility in water [133]. Taking these features into account, a 22 amino acids compound (ATI-5261) was synthesized by introducing negatively charged glutamine residues to a segment derived from the C-terminal domain of apoE at positions 14 and 19 together with hydrophobic acids (A, W, F, and L) [133]. This compound lacks tandem helical repeats and exhibits efficient stimulation of cholesterol efflux indicating that the ability to promote cholesterol efflux is not dependent on multiple *α*-helices as previously thought. ATI-5261 displays high *α*-helicity as shown by circular dichroism, hydrophobic moment, and increased solubility characteristics [132]. *In vitro*, ATI-5261 exerts its effects through ABCA1 in a fashion similar to that of HDL and successfully enhances cholesterol efflux from macrophages and reduces aortic atherosclerosis by up to 45% after intraperitoneal injection in mice [133]. ATI-526 increases faeces cholesterol transport in mice [133, 134]. The compound presently waits early phase clinical trials.

### 14.3. Endothelial Lipase Inhibitors (Figure 1, Part 11)

EL inhibition may represent potential future therapies to reduce apoA-I catabolism and to increase plasma apoA-I and HDL-C levels (Table 3). Human genetic studies have confirmed that variation of the EL gene is an important determinant of plasma HDL-C level [135]. However, how changes in HDL-C level attributed to EL may affect atherosclerosis is still not clear. Some human studies propose an atherogenic role for EL, with a positive association of plasma level of EL mass and coronary artery calcification [136]. Carrier of EL variants associated with HDL-C levels have been reported to have decreased risk of coronary artery disease [137], but this association has not been observed in other studies [64]. Mendelian randomization data casts doubt on whether genetic variability at the EL gene locus that increases HDL-C protects against cardiovascular diseases [31]. Studies in mice showed that EL overexpression reduces HDL-C and apo A-I levels [36, 138] due to increased renal catabolism. Conversely, gene deletion of EL results in increased HDL-C and apo A-I levels [139]. Although EL inactivation was expected to inhibit atherosclerosis by raising HDL-C, the effect of EL inactivation seems more complex than expected. Deficiency of EL resulted in an accumulation of small dense LDL, a potentially atherogenic mechanism. Brown et al. showed that targeted inactivation of EL increased plasma HDL-C level and inhibited atherosclerosis [140]. On the other hand, Ishida et al. have previously reported that targeted inactivation of EL increased plasma HDL-C level and inhibited atherosclerosis in apoE^−/−^ mice [138]. These findings initiated the synthesis of a library of small molecule inhibitors of EL based on sulfonylurea backbone [141]. Recently, many of new EL inhibitors synthesized from boronic acid were evaluated for potency against both EL and LPL [142]. The enthusiasm for EL inhibitors is somewhat tempered by Mendelian randomization data showing that variabilities at the EL and CETP genes that increase HDL-C are not associated with protection against CVD [143].

### 14.4. LCAT Modulators (Figure 1, Part 12)

Several drug development approaches have recently been started for modulating LCAT activity (Table 3). One strategy is being investigated is the use of engineered recombinant LCAT protein entity (rLCAT) as a replacement therapy in the case of familial LCAT deficiency. Early studies for treatment of atherosclerosis and CVD by raising HDL-C through plasma LCAT enzyme activity were initiated by Zhou et al. in a rabbit model [144]. Data concluded that recombinant LCAT administration may represent a novel approach for the treatment of atherosclerosis and the dyslipidemia associated with low HDL. A preclinical mouse study of human recombinant LCAT (rLCAT) was recently reported [145]. An rLCAT (rLCAT, AlphaCore Pharma, Ann Arbor, MI, USA) was injected into LCAT-null mice and found to reverse the abnormal lipoprotein profile by increasing HDL-C to near normal levels for several days. Intravenous infusion of human rLCAT in rabbits was found to raise HDL-C, to increase fecal secretion of cholesterol, and to reduce atherosclerosis [146]. Another potential alternative to LCAT injection for treatment of human LCAT deficiency was recently reported in which adipocytes transfected with LCAT were transplanted into mice and were found to raise HDL-C [147]. Only one LCAT modulator has reached early clinical development, ETC-642, but little data are available on the outcomes of treatment with this agent [148].

## 15. Apo A-I Upregulator (Figure 1, Part 3)

### 15.1. RVX-208

Reservelogix-208 (RVX-208) is a small molecule that increases endogenous synthesis of apoA-I (Table 3). RVX-208 has a molecular weight of roughly 400 Da belonging to the quinazoline family. Recently, in African green monkeys, oral administration of RVX-208 resulted in increased levels of plasma apoA-I and HDL-C [149]. Serum from these animals was shown to mediate enhanced cholesterol efflux from J774 macrophages via the ABCA1, ABCG1, and SR-BI-dependent pathways [149]. Serum from human subjects treated with RVX-208 exhibited increased cholesterol efflux capacity despite a relatively modest increase in HDL-C levels [150]. In a phase II trial, modest changes in HDL-C and apoA-I were reported in 299 statin-treated patients with stable CAD [151]. Two Phase 2b studies are ongoing, one involves 172 statin-treated patients randomized for RVX-208 100 mg or placebo twice daily for 24 weeks [152] and the other phase trial will investigate the effect of RVX-208 on coronary atherosclerosis assessed by intravascular ultrasound [153].

### 15.2. Synthetic Liver X Receptor (LXR) Agonists (Figure 1, Part 7)

Synthetic LXR agonists including LXR*α*/*β* are known to induce the transcription of ABCA1 and ABCG1 [53]. As potent activators of the cellular cholesterol efflux, these compounds have been shown to raise HDL-C levels and to reduce atherosclerosis in transgenic mouse models [154]. Thus, LXR agonist's activation may be a promising pharmacologic target for the treatment of dyslipidemia and atherosclerosis (Table 3). Unfortunately, development of first generation LXR compounds has been hampered by their induction of expression of lipogenic genes in the liver, which increase the levels of triglycerides and promote hepatic steatosis [53]. Various synthetically engineered LXR agonists have been developed and tested in animal models (T0901317, LXR-623, GW3965, GW6340, AZ876, and ATI-111). They all show high potency for interacting with LXR*α*/*β* receptors, but none of them shows selectivity for ABCA1 and ABCG1 [53, 155–157]. T091317 is a LXR activator, broadly used for research in LXR biology [158, 159]. T091317 consistently decreases atherosclerosis in mice models and induces gene expression of NCP1 and NCP2 in macrophages resulting in enriched cholesterol content in the outer layer of plasma membrane [158]. The LXR agonist LXR-623 is associated with increased expression of ABCA1 and ABCG1 in cells [160], but adverse central nervous system-related effects were noted in more than half of patients, leading to termination of the study [161]. In combination with simvastatin in a rabbit model of advanced atherosclerosis, LXR-623 showed a reduction in the rate of plaque progression [156]. Other agonists (AZ876 and GW3965) were shown to reduce the number of atherosclerotic lesions [159]. The LXR agonist GW6340, an intestine specific LXR*α*/*β* agonist, promoted macrophage specific cellular cholesterol efflux and increased intestinal excretion of HDL-derived cholesterol [155]. More recently, a novel synthetic LXR agonist, ATI-111, that is, more potent than T0901317, inhibited atherosclerotic progression and prevented atheromatous plaque formation in mice [157]. It is clear that research on more selective LXR ligands is an active field of experimental pharmacology. A new LXR inverse agonist (SR9238) was designed for nonalcoholic fatty disease to suppress hepatic steatosis [162]. Data from mouse model indicated that this strategy may hold promise in the treatment of liver disease [162].

### 15.3. Synthetic FXR Agonists (Figure 1, Part 9)

The Farnesoid X receptor (FXR) is a bile acid-activated nuclear receptor that plays an important role in the regulation of cholesterol and more specifically HDL homeostasis [163]. Preclinical studies showed that activation of FXR leads to both pro- and antiatherosclerosis and a major metabolic of FXR agonists observed consistently in animal studies was the reduction of plasma HDL [163, 164]. Hambruch et al. showed that FXR agonists induce the hepatic steps of HDL-derived cholesterol excretion into feces in mice and monkeys [164]. For these reasons, FXR agonists have received attention as a potential therapeutic target [165, 166], and different agonists have been generated as a strategy for HDL-C raising therapies. These include GW4064, 6-ECDCA, FXR-450, and PX20606 [163] (Table 3). Both synthetic FXR agonists FXR-450 and PX20606 demonstrated potent plasma cholesterol lowering in mice, whereas GW4064 and 6-ECDCA showed only limited effects [164]. GW4064 as previously demonstrated it has potential cell toxicity and uncertain bioavailability prevents its development for clinical studies [165]. Interestingly, in CETPtg-LDLR(^−/−^) mice PX20606 caused highly significant decrease in atherosclerotic plaque size despite the observed HDL-C reduction [164]. Data from normolipidemic monkeys treated with PX20606 significantly and specifically lowered the HDL2 subclass without changing apoA-I levels. In these studies, the basic mechanisms of FXR mediating HDL-C clearance are conserved in mice and monkeys. These observations will support further studies to investigate the potential roles of FXR activation on HDL species.

## 16. Gene Therapy

Gene therapy or somatic gene transfer for the treatment of severe lipoprotein disorders has been previously attempted, with limited success in homozygous familial hypercholesterolemia [166, 167]. Animal experiments with apoA-I transgenes have yielded beneficial results for the prevention of atherosclerosis [168, 169]. To date, this approach has little application in man. Recently, an adeno-associated virus-based treatment for lipoprotein lipase deficiency of enhanced activity variant of LPL(S447X) alipogene tiparvovec was tested [170, 171]. The LPLS447X variant is found in 20% of Caucasians and is associated with enhanced removal of proatherogenic apoB100-containing particles, including LDL-cholesterol [171], lower plasma TG levels, higher HDL cholesterol concentrations, and lower rates of cardiovascular disease, when compared to the general population. This data provided the rationale for a gene therapy trial in man. Alipogene tiparvovec (AAV1-LPLS447X) therapy in patients with severe hypertriglyceridemia secondary to LPL deficiency and recurrent pancreatitis was performed in 20 subjects. Triglyceride levels fell in 40% of the subjects. Unfortunately, the authors do not report HDL-C levels in these patients. However, this serves as a proof of concept for gene therapy in man. Alipogene tiparvovec was recently discontinued because of unfavorable opinion about its risk-benefit given by the European Medicines Evaluation Agency. Animal data supports novel gene-based approaches to increase HDL-C. A potential mechanism to increase of HDL cholesterol is increasing ABCA1 and ABCG1 expression through the microRNA (inhibition of miR-33 being the most promising candidate to date) (Figure 1, part 6). Overexpression of antisense miR-33 using lentivirus in mice showed a 50% increase in hepatic ABCA1 protein levels and a concomitant 25% increase in plasma HDL levels after 6 days [51]. Marquart et al. showed that injection of an anti-miR-33 oligonucleotide in mice resulted in a substantial increase in ABCA1 expression and HDL levels [172]. Furthermore, it was shown in mice that miR-33 resulted in decreased cholesterol efflux [173] which provide a potentially novel therapeutic approach. These data suggest that miR-33 might be a possible target for the treatment of cardiovascular and metabolic disorders.

## 17. Concluding Remarks 

Potential new therapeutic treatment of atherosclerosis based on HDL biology remains in preclinical steps. Strategies to raise HDL pharmacologically have, so far, not yielded sufficiently positive results to make firm clinical treatment recommendation. The concept that HDL-C is a risk factor (i.e., having a direct, causal role in atherosclerosis) has been challenged (Table 2). It is conceivable that HDL-C may simply be a biomarker of cardiovascular health. Moreover, the recent HSP2-THRIVE and dal-OUTCOMES studies put the HDL-C hypothesis in question. The association between HDL and CVD is more complex than previously thought and is probably mediated by various HDL functions, such as reverse cholesterol transport or anti-inflammatory, antioxidant, and vascular protective properties. It may be possible also that HDL-C level is correlated with other nonmeasured active subfractions that may act directly to reduce and predict CVD risk. To better understand the relation between HDL and atherosclerosis, we should consider developing and measuring better markers of HDL function, rather than the cholesterol mass within HDL particles.

## Figures and Tables

**Table 1:** Characteristics of pathways for cholesterol efflux from cells to plasma.

Characteristics	Aqueous diffusion	SR-BI	ABCA1	ABCG1
Energy requirement	Passive	Passive	Active	Active
Cholesterol flux	Bidirectional	Bidirectional	Unidirectional	Unidirectional
Preferred HDL acceptors	HDL2, HDL3	HDL2, HDL3	pre*β*-HDL Lipid-poor/free apoA-I	HDL2, HDL3

**Table 2:** Controversies surrounding the “HDL hypothesis.”

HDL and atherosclerosis: pro	The “HDL hypothesis” questioned
Strong biological plausibility for HDL as a therapeutic target	Mendelian Randomization does not support HDL-cholesterol as a causal risk factor

The epidemiological association between HDL-C and CVD is strong and coherent	HDL-C loses its predictive value if LDL-C is low

Animal data is unequivocal: HDL protects against atherosclerosis	The clinical trial data with drugs that raise HDL-C (fibrates, niacin, torcetrapib, and dalcetrapib) is neutral

**Table 3:** Summary of selected strategies to increase HDL/apoA-I and potential compound under development. Numbers in the right hand column refer to Figure 1.

Pharmacotherapeutic Strategy	Drug	Aim	Figure 1
Recombinant apoA-I Milano/phospholipids	ETC-216	Directly augmenting apoA-I/HDL pool	1
Purified native apoA-I/phospholipids	CSL-111 CSL-112	Directly augmenting apoA-I/HDL pool	2
Upregulators of endogenous apoA-I production	RVX-208	Directly augmenting apoA-I/HDL pool	3
ApoA-I mimetic peptides	D-4F L-4F 6F, 5A ATI-5261	Mimicking apoA-I functionality	4
Autologous Delipidated HDL	Selective HDL delipidated	Directly augmenting apoA-I/HDL pool	5
Gene therapy	miR-33	Modulating HDL levels and cholesterol efflux expression	6
Liver X receptor agonists	LXR*α*/*β* agonists LxR-623 T0901317, GW3965 ATI-111	Enhancing RCT & Macrophage cholesterol efflux	7
Niacin receptor agonists	ARI-3037MO	Indirectly augmenting apoA-I and HDL-cholesterol	8
Farnesoid X receptor	FxR-450	Modulate HDL levels	9
Cholesteryl ester transfer inhibitors	Anacetrapib MK-0859Evacetrapib LY248595	Indirectly augmenting apoA-I and HDL-cholesterol	10
Endothelial lipase inhibition	Boronic acid inhibitors Selective sulfonylfuran urea	Increasing HDL-cholesterol	11
LCAT activators	rLCAT ETC-642	Enhancing RCT	12

## Abbreviations

ABCA1: ATP-binding cassette transporter A1
ABCG1: ATP-binding cassette transporter G1
ApoA-I: Apolipoprotein A-I
ApoB: Apolipoprotein B
ApoE: Apolipoprotein E
CETP: Cholesterol ester transfer protein
CVD: Coronary vascular disease
FC: Free cholesterol
FXR: Farnesoid X-receptor
HDL: High-density lipoprotein
HDL-C: High-density lipoprotein-cholesterol
LCAT: Lecithin-cholesterol acyltransferase
LDL: Low-density lipoprotein
LXR: Liver X-receptor
ox-LDL: Oxidized low-density lipoprotein
RCT: Reverse cholesterol transport
RXR: Retinoid X-receptor
SR-B1: Scavenger-receptor B-I
TG: Triglyceride
UTC: Ultracentrifugation.

## References

[1] Rader DJ, deGoma EM (2012). Approach to the patient with extremely low HDL-cholesterol. *The Journal of Clinical Endocrinology & Metabolism*.

[2] Rader DJ, Tall AR (2012). The not-so-simple HDL story: is it time to revise the HDL cholesterol hypothesis?. *Nature Medicine*.

[3] Baigent C, Blackwell L, Emberson J (2010). Efficacy and safety of more intensive lowering of LDL cholesterol: a meta-analysis of data from 170,000 participants in 26 randomised trials. *The Lancet*.

[4] Boden WE, Probstfield JL, Anderson T (2011). Niacin in patients with low HDL cholesterol levels receiving intensive statin therapy. *New England Journal of Medicine*.

[5] Rosenson RS, Gotto Jr. AM (2013). When clinical trials fail to address treatment gaps: the failure of niacin-laropiprant to reduce cardiovascular events. *Current Atherosclerosis Reports*.

[6] Besler C, Heinrich K, Rohrer L (2011). Mechanisms underlying adverse effects of HDL on eNOS-activating pathways in patients with coronary artery disease. *Journal of Clinical Investigation*.

[7] Rosenson RS, Brewer HB, Chapman MJ (2011). HDL measures, particle heterogeneity, proposed nomenclature, and relation to atherosclerotic cardiovascular events. *Clinical Chemistry*.

[8] Niesor EJ (2011). Different effects of compounds decreasing cholesteryl ester transfer protein activity on lipoprotein metabolism. *Current Opinion in Lipidology*.

[9] Delalla OF, Elliot HA, Gofman JW (1954). Ultracentrifugal studies of high density serum lipoproteins in clinically healthy adults. *The American Journal of Physiology*.

[10] Fruchart JC, Bard JM (1991). Lipoprotein particle measurement: an alternative approach to classification of lipid disorders. *Current Opinion in Lipidology*.

[11] Camont L, Chapman MJ, Kontush A (2011). Biological activities of HDL subpopulations and their relevance to cardiovascular disease. *Trends in Molecular Medicine*.

[12] Heinecke JW (2009). The HDL proteome: a marker—and perhaps mediator—of coronary artery disease. *Journal of Lipid Research*.

[13] Davidson WS, Silva RAGD, Chantepie S, Lagor WR, Chapman MJ, Kontush A (2009). Proteomic analysis of defined hdl subpopulations reveals particle-specific protein clusters: relevance to antioxidative function. *Arteriosclerosis, Thrombosis, and Vascular Biology*.

[14] Superko HR, Pendyala L, Williams PT, Momary KM, King SB, Garrett BC (2012). High-density lipoprotein subclasses and their relationship to cardiovascular disease. *Journal of Clinical Lipidology*.

[15] Khera AV, Rader DJ (2010). Future therapeutic directions in reverse cholesterol transport. *Current Atherosclerosis Reports*.

[16] Ansell BJ, Navab M, Hama S (2003). Inflammatory/antiinflammatory properties of high-density lipoprotein distinguish patients from control subjects better than high-density lipoprotein cholesterol levels and are favorably affected by simvastatin treatment. *Circulation*.

[17] Di Angelantonio E, Sarwar N, Perry P (2009). Major lipids, apolipoproteins, and risk of vascular disease. *Journal of the American Medical Association*.

[18] Mackey RH, Greenland P, Goff DC, Lloyd-Jones D, Sibley CT, Mora S (2012). High-density lipoprotein cholesterol and particle concentrations, carotid atherosclerosis, and coronary events: MESA (multi-ethnic study of atherosclerosis). *Journal of the American College of Cardiology*.

[19] Ginsberg HN, Elam MB, Lovato LC (2010). Effects of combination lipid therapy in type 2 diabetes mellitus. *New England Journal of Medicine*.

[20] Jun M, Foote C, Lv J (2010). Effects of fibrates on cardiovascular outcomes: a systematic review and meta-analysis. *The Lancet*.

[21] Fruchart JC, Sacks F, Hermans MP (2008). The residual risk reduction initiative: a call to action to reduce residual vascular risk in patients with dyslipidemia. *American Journal of Cardiology*.

[22] Arsenault BJ, Barter P, DeMicco DA (2011). TNT Study Investigators. Prediction of cardiovascular events in statin-treated stable coronary patients by lipid and nonlipid biomarkers. *Journal of the American College of Cardiology*.

[23] van der Steeg WA, Holme I, Boekholdt SM (2008). High-density lipoprotein cholesterol, high-density lipoprotein particle size, and apolipoprotein A-I: significance for cardiovascular risk: the IDEAL and EPIC-Norfolk studies. *Journal of the American College of Cardiology*.

[24] Barter P (2009). Lessons Learned from the Investigation of Lipid Level Management to Understand its Impact in Atherosclerotic Events (ILLUMINATE) Trial. *American Journal of Cardiology*.

[25] Barter PJ, Caulfield M, Eriksson M (2007). ILLUMINATE Investigators. Effects of torcetrapib in patients at high risk for coronary events. *New England Journal of Medicine*.

[26] Ridker PM, Genest J, Boekholdt SM (2010). HDL cholesterol and residual risk of first cardiovascular events after treatment with potent statin therapy: an analysis from the JUPITER trial. *The Lancet*.

[27] Schwartz GG, Olsson AG, Abt M (2012). dal-OUTCOMES Investigators. Effects of dalcetrapib in patients with a recent acute coronary syndrome. *New England Journal of Medicine*.

[28] HPS2-THRIVE Collaborative Group (2013). HPS2-THRIVE randomized placebo-controlled trial in 25 673 high-risk patients of ER niacin/laropiprant: trial design, pre-specified muscle and liver outcomes, and reasons for stopping study treatment. *European Heart Journal*.

[29] Cook C, Sheets C (2011). Clinical equipoise and personal equipoise: two necessary ingredients for reducing bias in manual therapy trials. *Journal of Manual and Manipulative Therapy*.

[30] Frikke-Schmidt R, Nordestgaard BG, Schnohr P, Tybjærg-Hansen A (2004). Single nucleotide polymorphism in the low-density lipoprotein receptor is associated with a threefold risk of stroke: a case-control and prospective study. *European Heart Journal*.

[31] Voight BF, Peloso GM, Orho-Melander M (2012). Plasma HDL cholesterol and risk of myocardial infarction: a mendelian randomisation study. *The Lancet*.

[32] Glomset JA (1968). The plasma lecithins:cholesterol acyltransferase reaction. *Journal of Lipid Research*.

[33] Devries-Seimon T, Li Y, Pin MY (2005). Cholesterol-induced macrophage apoptosis requires ER stress pathways and engagement of the type A scavenger receptor. *Journal of Cell Biology*.

[34] Khera AV, Cuchel M, De La Llera-Moya M (2011). Cholesterol efflux capacity, high-density lipoprotein function, and atherosclerosis. *New England Journal of Medicine*.

[35] Hassan HH, Denis M, Lee DYD (2007). Identification of an ABCA1-dependent phospholipid-rich plasma membrane apolipoprotein A-I binding site for nascent HDL formation: implications for current models of HDL biogenesis. *Journal of Lipid Research*.

[36] Rosenson RS, Brewer HB, Davidson WS (2012). Cholesterol efflux and atheroprotection: advancing the concept of reverse cholesterol transport. *Circulation*.

[37] Rothblat GH, Phillips MC (2010). High-density lipoprotein heterogeneity and function in reverse cholesterol transport. *Current Opinion in Lipidology*.

[38] Adorni MP, Zimetti F, Billheimer JT (2007). The roles of different pathways in the release of cholesterol from macrophages. *Journal of Lipid Research*.

[39] Oram JF, Vaughan AM (2000). ABCA1-mediated transport of cellular cholesterol and phospholipids to HBL apolipoproteins. *Current Opinion in Lipidology*.

[40] Nagao K, Tomioka M, Ueda K (2011). Function and regulation of ABCA1—membrane meso-domain organization and reorganization. *FEBS Journal*.

[41] Zhao Y, Van Berkel TJC, Van Eck M (2010). Relative roles of various efflux pathways in net cholesterol efflux from macrophage foam cells in atherosclerotic lesions. *Current Opinion in Lipidology*.

[42] Sorci-Thomas MG, Owen JS, Fulp B (2012). Nascent high density lipoproteins formed by ABCA1 resemble lipid rafts and are structurally organized by three apoA-I monomers. *Journal of Lipid Research*.

[43] Phillips MC (2012). New insights into the determination of HDL structure by apolipoproteins. *Journal of Lipid Research*.

[44] Okuhira K, Fitzgerald ML, Tamehiro N (2010). Binding of PDZ-RhoGEF to ATP-binding cassette transporter A1 (ABCA1) induces cholesterol efflux through RhoA activation and prevention of transporter degradation. *Journal of Biological Chemistry*.

[45] Neufeld EB, Remaley AT, Demosky SJ (2001). Cellular localization and trafficking of the human ABCA1 transporter. *Journal of Biological Chemistry*.

[46] Kellner-Weibel G, de la Llera-Moya M (2011). Update on HDL receptors and cellular cholesterol transport. *Current Atherosclerosis Reports*.

[47] Timmins JM, Lee JY, Boudyguina E (2005). Targeted inactivation of hepatic Abca1 causes profound hypoalphalipoproteinemia and kidney hypercatabolism of apoA-I. *Journal of Clinical Investigation*.

[48] Brunham LR, Kruit JK, Iqbal J (2006). Intestinal ABCA1 directly contributes to HDL biogenesis in vivo. *Journal of Clinical Investigation*.

[49] Zhang Y, McGillicuddy FC, Hinkle CC (2010). Adipocyte modulation of high-density lipoprotein cholesterol. *Circulation*.

[50] Fernández-Hernando C, Suárez Y, Rayner KJ, Moore KJ (2011). MicroRNAs in lipid metabolism. *Current Opinion in Lipidology*.

[51] Rayner KJ, Sheedy FJ, Esau CC (2011). Antagonism of miR-33 in mice promotes reverse cholesterol transport and regression of atherosclerosis. *Journal of Clinical Investigation*.

[52] Rayner KJ, Esau CC, Hussain FN (2011). Inhibition of miR-33a/b in non-human primates raises plasma HDL and lowers VLDL triglycerides. *Nature*.

[53] Oosterveer MH, Grefhorst A, Groen AK, Kuipers F (2010). The liver X receptor: control of cellular lipid homeostasis and beyond: implications for drug design. *Progress in Lipid Research*.

[54] De La Llera-Moya M, Drazul-Schrader D, Asztalos BF, Cuchel M, Rader DJ, Rothblat GH (2010). The ability to promote efflux via ABCA1 determines the capacity of serum specimens with similar high-density lipoprotein cholesterol to remove cholesterol from macrophages. *Arteriosclerosis, Thrombosis, and Vascular Biology*.

[55] Akiyama TE, Sakai S, Lambert G (2002). Conditional disruption of the peroxisome proliferator-activated receptor *γ* gene in mice results in lowered expression of ABCA1, ABCG1, and apoE in macrophages and reduced cholesterol efflux. *Molecular and Cellular Biology*.

[56] Rayner KJ, Suلrez Y, Dلvalos A (2010). MiR-33 contributes to the regulation of cholesterol homeostasis. *Science*.

[57] Gelissen IC, Cartland S, Brown AJ (2010). Expression and stability of two isoforms of ABCG1 in human vascular cells. *Atherosclerosis*.

[58] Kerr ID, Haider AJ, Gelissen IC (2011). The ABCG family of membrane-associated transporters: you don't have to be big to be mighty. *British Journal of Pharmacology*.

[59] Tarling EJ, Bojanic DD, Tangirala RK (2010). Impaired development of atherosclerosis in Abcg1-/- Apoe -/- mice: identification of specific oxysterols that both accumulate in Abcg1-/- Apoe-/- tissues and induce apoptosis. *Arteriosclerosis, Thrombosis, and Vascular Biology*.

[60] Kennedy MA, Barrera GC, Nakamura K (2005). ABCG1 has a critical role in mediating cholesterol efflux to HDL and preventing cellular lipid accumulation. *Cell Metabolism*.

[61] Yvan-Charvet L, Wang N, Tall AR (2010). Role of HDL, ABCA1, and ABCG1 transporters in cholesterol efflux and immune responses. *Arteriosclerosis, Thrombosis, and Vascular Biology*.

[62] Van Eck M, Bos IST, Hildebrand RB, Van Rij BT, Van Berkel TJC (2004). Dual role for scavenger receptor class B, type I on bone marrow-derived cells in atherosclerotic lesion development. *American Journal of Pathology*.

[63] Demetz E, Tancevski I, Duwensee K (2012). Inhibition of hepatic scavenger receptor-class B type I by RNA interference decreases atherosclerosis in rabbits. *Atherosclerosis*.

[64] Vergeer M, Korporaal SJA, Franssen R (2011). Genetic variant of the scavenger receptor BI in humans. *New England Journal of Medicine*.

[65] Al-Jarallah A, Trigatti BL (2010). A role for the scavenger receptor, class B type I in high density lipoprotein dependent activation of cellular signaling pathways. *Biochimica et Biophysica Acta*.

[66] Wang X, Collins HL, Ranalletta M (2007). Macrophage ABCA1 and ABCG1, but not SR-BI, promote macrophage reverse cholesterol transport in vivo. *Journal of Clinical Investigation*.

[67] Hildebrand RB, Lammers B, Meurs I (2010). Restoration of high-density lipoprotein levels by cholesteryl ester transfer protein expression in scavenger receptor class B Type i (SR-BI) knockout mice does not normalize pathologies associated with SR-BI deficiency. *Arteriosclerosis, Thrombosis, and Vascular Biology*.

[68] El Bouhassani M, Gilibert S, Moreau M (2011). Cholesteryl ester transfer protein expression partially attenuates the adverse effects of SR-BI receptor deficiency on cholesterol metabolism and atherosclerosis. *Journal of Biological Chemistry*.

[69] Masson D, Koseki M, Ishibashi M (2009). Increased HDL cholesterol and ApoA-I in humans and mice treated with a novel SR-BI inhibitor. *Arteriosclerosis, Thrombosis, and Vascular Biology*.

[70] Navab M, Reddy ST, Van Lenten BJ, Fogelman AM (2011). HDL and cardiovascular disease: atherogenic and atheroprotective mechanisms. *Nature Reviews Cardiology*.

[71] Besler C, Lüscher TF, Landmesser U (2012). Molecular mechanisms of vascular effects of High-density lipoprotein: alterations in cardiovascular disease. *EMBO Molecular Medicine*.

[72] Kontush A, Chapman MJ (2010). Antiatherogenic function of HDL particle subpopulations: focus on antioxidative activities. *Current Opinion in Lipidology*.

[73] Sorrentino SA, Besler C, Rohrer L (2010). Endothelial-vasoprotective effects of high-density lipoprotein are impaired in patients with type 2 diabetes mellitus but are improved after extended-release niacin therapy. *Circulation*.

[74] Navab M, Hama SY, Hough GP, Subbanagounder G, Reddy ST, Fogelman AM (2001). A cell-free assay for detecting HDL that is dysfunctional in preventing the formation of or inactivating oxidized phospholipids. *Journal of Lipid Research*.

[75] Liu Y, Tang C (2012). Regulation of ABCA1 functions by signaling pathways. *Biochimica et Biophysica Acta*.

[76] Patel S, Di Bartolo BA, Nakhla S (2010). Anti-inflammatory effects of apolipoprotein A-I in the rabbit. *Atherosclerosis*.

[77] Riwanto M, Rohrer L, Roschitzki B (2013). Altered activation of endothelial anti- and proapoptotic pathways by high-density lipoprotein from patients with coronary artery disease: role of high-density lipoprotein-proteome remodeling. *Circulation*.

[78] Sato K, Okajima F (2010). Role of sphingosine 1-phosphate in anti-atherogenic actions of high-density lipoprotein. *World Journal of Biological Chemistry*.

[79] Alwaili K, Bailey D, Awan Z (2012). The HDL proteome in acute coronary syndromes shifts to an inflammatory profile. *Biochimica et Biophysica Acta*.

[80] Mineo C, Yuhanna IS, Quon MJ, Shaul PW (2003). High density lipoprotein-induced endothelial nitric-oxide synthase activation is mediated by Akt and MAP kinases. *Journal of Biological Chemistry*.

[81] Suc I, Escargueil-Blanc I, Troly M, Salvayre R, Negre-Salvayre A (1997). HDL and apoA prevent cell death of endothelial cells induced by oxidized LDL. *Arteriosclerosis, Thrombosis, and Vascular Biology*.

[82] Langmann T, Klucken J, Reil M (1999). Molecular cloning of the human ATP-Binding cassette transporter 1 (hABC1): evidence for sterol-dependent regulation in macrophages. *Biochemical and Biophysical Research Communications*.

[83] Nofer JR, Brodde MF, Kehrel BE (2010). High-density lipoproteins, platelets and the pathogenesis of atherosclerosis: frontiers in research review: physiological and pathological functions of high-density lipoprotein. *Clinical and Experimental Pharmacology and Physiology*.

[84] Li Y, Dong JB, Wu MP (2008). Human ApoA-I overexpression diminishes LPS-induced systemic inflammation and multiple organ damage in mice. *European Journal of Pharmacology*.

[85] Figueirêdo PMS, Catani CF, Yano T (2003). Serum high-density lipoprotein (HDL) inhibits in vitro enterohemolysin (EHly) activity produced by enteropathogenic Escherichia coli. *FEMS Immunology and Medical Microbiology*.

[86] Yin K, Tang SL, Yu XH (2013). Apolipoprotein A-I inhibits LPS-induced atherosclerosis in ApoE-/- mice possibly via activated STAT3-mediated upregulation of tristetraprolin. *Acta Pharmacologica Sinica*.

[87] Hager KM, Hajduk SL (1997). Mechanism of resistance of African trypanosomes to cytotoxic human HDL. *Nature*.

[88] Krishna R, Anderson MS, Bergman AJ (2007). Effect of the cholesteryl ester transfer protein inhibitor, anacetrapib, on lipoproteins in patients with dyslipidaemia and on 24-h ambulatory blood pressure in healthy individuals: two double-blind, randomised placebo-controlled phase I studies. *The Lancet*.

[89] Chapman MJ, Le Goff W, Guerin M, Kontush A (2010). Cholesteryl ester transfer protein: at the heart of the action of lipid-modulating therapy with statins, fibrates, niacin, and cholesteryl ester transfer protein inhibitors. *European Heart Journal*.

[90] Cannon CP, Shah S, Dansky HM (2010). Safety of anacetrapib in patients with or at high risk for coronary heart disease. *New England Journal of Medicine*.

[91] Lauring B, Taggart AK, Tata JR (2012). Niacin lipid efficacy is independent of both the niacin receptor GPR109A and free fatty acid suppression. *Science Translational Medicine*.

[92] http://clinicaltrials.gov/ct2/show/NCT01252953.

[93] Nicholls SJ, Brewer HB, Kastelein JJ (2011). Effects of the CETP inhibitor evacetrapib administered as monotherapy or in combination with statins on HDL and LDL cholesterol: a randomized controlled trial. *Journal of the American Medical Association*.

[94] http://clinicaltrials.gov/show/NCT01687998.

[95] Carlson LA (2005). Nicotinic acid: the broad-spectrum lipid drug. A 50th anniversary review. *Journal of Internal Medicine*.

[96] Wierzbicki AS (2011). Niacin: the only vitamin that reduces cardiovascular events. *International Journal of Clinical Practice*.

[97] (1975). Clofibrate and niacin in coronary heart disease. *Journal of the American Medical Association*.

[98] Bruckert E, Labreuche J, Amarenco P (2010). Meta-analysis of the effect of nicotinic acid alone or in combination on cardiovascular events and atherosclerosis. *Atherosclerosis*.

[99] Cannon CP (2011). High-density lipoprotein cholesterol as the Holy Grail. *Journal of the American Medical Association*.

[100] Gouni-Berthold I, Berthold HK (2013). The role of Niacin in lipid-lowering treatment: are we aiming too high?. *Current Pharmaceutical Design*.

[101] Parson HK, Harati H, Cooper D, Vinik AI (2013). The role of prostaglandin D2 and the autonomic nervous system on Niacin induced flushing. *Journal of Diabetes*.

[102] Bays HE, Shah A, Dong Q, McCrary Sisk C, Maccubbin D (2011). Extended-release niacin/laropiprant lipid-altering consistency across patient subgroups. *International Journal of Clinical Practice*.

[103] http://www.arisaph.com/newsroom/press.php.

[104] Sirtori CR, Calabresi L, Franceschini G (2001). Cardiovascular status of carriers of the apolipoprotein A-IMilano mutant: the limone sul garda study. *Circulation*.

[105] Nissen SE, Tsunoda T, Tuzcu EM (2003). Effect of recombinant ApoA-I Milano on coronary atherosclerosis in patients with acute coronary syndromes: a randomized controlled trial. *Journal of the American Medical Association*.

[106] http://www.faqs.org/sec-filings/091224/MEDICINES-CO-DE_8-K.

[107] Tardif JC (2010). Emerging high-density lipoprotein infusion therapies: fulfilling the promise of epidemiology?. *Journal of Clinical Lipidology*.

[108] Ibanez B, Giannarelli C, Cimmino G (2012). Recombinant HDL(Milano) exerts greater anti-inflammatory and plaque stabilizing properties than HDL(wild-type). *Atherosclerosis*.

[109] Chenevard R, Hürlimann D, Spieker L (2012). Reconstituted HDL in acute coronary syndromes. *Cardiovascular Therapeutics*.

[110] A single ascending dose study examining the safety and pharmacokinetic profile of reconstituted high density lipoprotein (CSL112) administered to patients. In: ClinicalTrials.gov. National Library of Medicine. http://www.clinicaltrials.gov/ct2/show/NCT01499420.

[111] Waksman R, Torguson R, Kent KM (2010). A first-in-man, randomized, placebo-controlled study to evaluate the safety and feasibility of autologous delipidated high-density lipoprotein plasma infusions in patients with acute coronary syndrome. *Journal of the American College of Cardiology*.

[112] Sacks FM, Rudel LL, Conner A (2009). Selective delipidation of plasma HDL enhances reverse cholesterol transport in vivo. *Journal of Lipid Research*.

[113] Anantharamaiah GM, Jones JL, Brouillette CG (1985). Studies of synthetic peptide analogs of the amphiphatic helix. Structure of complexes with dimyristoyl phosphatidylcholine. *Journal of Biological Chemistry*.

[114] Smith JD (2010). Apolipoprotein A-I and its mimetics for the treatment of atherosclerosis. *Current Opinion in Investigational Drugs*.

[115] Datta G, Chaddha M, Hama S (2001). Effects of increasing hydrophobicity on the physical-chemical and biological properties of a class A amphipathic helical peptide. *Journal of Lipid Research*.

[116] Shah PK, Chyu KY (2005). Apolipoprotein A-I mimetic peptides: potential role in atherosclerosis management. *Trends in Cardiovascular Medicine*.

[117] Weihrauch D, Xu H, Shi Y (2007). Effects of D-4F on vasodilation, oxidative stress, angiostatin, myocardial inflammation, and angiogenic potential in tight-skin mice. *American Journal of Physiology*.

[118] Bloedon LT, Dunbar R, Duffy D (2008). Safety, pharmacokinetics, and pharmacodynamics of oral apoA-I mimetic peptide D-4F in high-risk cardiovascular patients. *Journal of Lipid Research*.

[119] Sherman CB, Peterson SJ, Frishman WH (2010). Apolipoprotein A-I mimetic peptides: a potential new therapy for the prevention of atherosclerosis. *Cardiology in Review*.

[120] Navab M, Shechter I, Anantharamaiah GM, Reddy ST, Van Lenten BJ, Fogelman AM (2010). Structure and function of HDL mimetics. *Arteriosclerosis, Thrombosis, and Vascular Biology*.

[121] Watson CE, Weissbach N, Kjems L (2011). Treatment of patients with cardiovascular disease with L-4F, an apo-A1 mimetic, did not improve select biomarkers of HDL function. *Journal of Lipid Research*.

[122] Ou J, Ou Z, Jones DW (2003). L-4F, an apolipoprotein A-1 mimetic, dramatically improves vasodilation in hypercholesterolemia and sickle cell disease. *Circulation*.

[123] Chen X, Burton C, Song X (2009). An apoa-I mimetic peptide increases LCAT activity in mice through increasing HDL concentration. *International Journal of Biological Sciences*.

[124] Navab M, Reddy ST, Anantharamaiah GM (2011). Intestine may be a major site of action for the apoA-I mimetic peptide 4F whether administered subcutaneously or orally. *Journal of Lipid Research*.

[125] Navab M, Reddy ST, Anantharamaiah GM (2012). D-4F-mediated reduction in metabolites of arachidonic and linoleic acids in the small intestine is associated with decreased inflammation in lowdensity lipoprotein receptor-null mice. *Journal of Lipid Research*.

[126] Remaley AT, Thomas F, Stonik JA (2003). Synthetic amphipathic helical peptides promote lipid efflux from cells by an ABCA1-dependent and an ABCA1-independent pathway. *Journal of Lipid Research*.

[127] Anantharamaiah GM, Mishra VK, Garber DW (2007). Structural requirements for antioxidative and anti-inflammatory properties of apolipoprotein A-I mimetic peptides. *Journal of Lipid Research*.

[128] Chattopadhyay A, Navab M, Hough G (2013). A novel approach to oral ApoA-I mimetic therapy. *Journal of Lipid Research*.

[129] Imaizumi S, Navab M, Morgantini C (2011). Dysfunctional high-density lipoprotein and the potential of apolipoprotein A-1 mimetic peptides to normalize the composition and function of lipoproteins. *Circulation Journal*.

[130] Tabet F, Remaley AT, Segaliny AI (2010). The 5A apolipoprotein A-I mimetic peptide displays antiinflammatory and antioxidant properties in vivo and in vitro. *Arteriosclerosis, Thrombosis, and Vascular Biology*.

[131] Carballo-Jane E, Chen Z, O’Neill E (2010). ApoA-I mimetic peptides promote pre-*β* HDL formation in vivo causing remodeling of HDL and triglyceride accumulation at higher dose. *Bioorganic and Medicinal Chemistry*.

[132] Zheng Y, Patel AB, Narayanaswami V, Hura GL, Hang B, Bielicki JK (2011). HDL mimetic peptide ATI-5261 forms an oligomeric assembly in solution that dissociates to monomers upon dilution. *Biochemistry*.

[133] Bielicki JK, Zhang H, Cortez Y (2010). A new HDL mimetic peptide that stimulates cellular cholesterol efflux with high efficiency greatly reduces atherosclerosis in mice. *Journal of Lipid Research*.

[134] http://circ.ahajournals.org/cgi/content/meeting_abstract/120/18_MeetingAbstracts/S445-a.

[135] Edmondson AC, Brown RJ, Kathiresan S (2009). Loss-of-function variants in endothelial lipase are a cause of elevated HDL cholesterol in humans. *The Journal of Clinical Investigation*.

[136] Badellino KO, Wolfe ML, Reilly MP, Rader DJ (2006). Endothelial lipase concentrations are increased in metabolic syndrome and associated with coronary atherosclerosis. *PLoS Medicine*.

[137] Tang NP, Wang LS, Yang L (2008). Protective effect of an endothelial lipase gene variant on coronary artery disease in a Chinese population. *Journal of Lipid Research*.

[138] Ishida T, Choi S, Kundu RK (2003). Endothelial lipase is a major determinant of HDL level. *Journal of Clinical Investigation*.

[139] deGoma EM, Rader DJ (2011). Novel HDL-directed pharmacotherapeutic strategies. *Nature Reviews Cardiology*.

[140] Brown RJ, Lagor WR, Sankaranaravanan S (2010). Impact of combined deficiency of hepatic lipase and endothelial lipase on the metabolism of both high-density lipoproteins and apolipoprotein b-containing lipoproteins. *Circulation Research*.

[141] Goodman KB, Bury MJ, Cheung M (2009). Discovery of potent, selective sulfonylfuran urea endothelial lipase inhibitors. *Bioorganic & Medicinal Chemistry Letters*.

[142] O'Connell DP, LeBlanc DF, Cromley D, Billheimer J, Rader DJ, Bachovchin WW (2012). Design and synthesis of boronic acid inhibitors of endothelial lipase. *Bioorganic & Medicinal Chemistry Letters*.

[143] Teslovich TM, Musunuru K, Smith AV (2010). Biological, clinical and population relevance of 95 loci for blood lipids. *Nature*.

[144] http://circ.ahajournals.org/cgi/content/meeting_abstract/120/18_MeetingAbstracts/S1175-b.

[145] Rousset X, Vaisman B, Auerbach B (2010). Effect of recombinant human lecithin cholesterol acyltransferase infusion on lipoprotein metabolism in mice. *Journal of Pharmacology and Experimental Therapeutics*.

[146] Rousset X, Shamburek R, Vaisman B, Amar M, Remaley AT (2011). Lecithin cholesterol acyltransferase: an anti- or pro-atherogenic factor?. *Current Atherosclerosis Reports*.

[147] Asada S, Kuroda M, Aoyagi Y (2011). Ceiling culture-derived proliferative adipocytes retain high adipogenic potential suitable for use as a vehicle for gene transduction therapy. *American Journal of Physiology*.

[148] Rader DJ (2006). Molecular regulation of HDL metabolism and function: implications for novel therapies. *Journal of Clinical Investigation*.

[149] Bailey D, Jahagirdar R, Gordon A (2010). RVX-208: a small molecule that increases apolipoprotein A-I and high-density lipoprotein cholesterol in vitro and in vivo. *Journal of the American College of Cardiology*.

[150] Gordon A, Jahagirdar R, Johannson J RVX-208 a small molecule that induces apolipoprotein A-I production progresses to phase Ib/IIa clinical trials.

[151] Nicholls SJ, Gordon A, Johansson J (2011). Efficacy and safety of a novel oral inducer of apolipoprotein A-I synthesis in statin-treated patients with stable coronary artery disease: a randomized controlled trial. *Journal of the American College of Cardiology*.

[152] Nicholls SJ, Gordon A, Johannson J (2012). ApoA-I induction as a potential cardioprotective strategy: rationale for the SUSTAIN and ASSURE studies. *Cardiovascular Drugs and Therapy*.

[153] Shah PK (2011). Atherosclerosis: targeting endogenous apo A-I-a new approach for raising HDL. *Nature Reviews Cardiology*.

[154] Lo Sasso G, Murzilli S, Salvatore L (2010). Intestinal specific LXR activation stimulates reverse cholesterol transport and protects from atherosclerosis. *Cell Metabolism*.

[155] Yasuda T, Grillot D, Billheimer JT (2010). Tissue-specific liver X receptor activation promotes macrophage reverse cholesterol transport in vivo. *Arteriosclerosis, Thrombosis, and Vascular Biology*.

[156] Giannarelli C, Cimmino G, Connolly TM (2012). Synergistic effect of liver X receptor activation and simvastatin on plaque regression and stabilization: an magnetic resonance imaging study in a model of advanced atherosclerosis. *European Heart Journal*.

[157] Peng D, Hiipakka RA, Xie JT (2011). A novel potent synthetic steroidal liver X receptor agonist lowers plasma cholesterol and triglycerides and reduces atherosclerosis in LDLR-/- mice. *British Journal of Pharmacology*.

[158] Rigamonti E, Helin L, Lestavel S (2005). Liver X receptor activation controls intracellular cholesterol trafficking and esterification in human macrophages. *Circulation Research*.

[159] Bradley MN, Hong C, Chen M (2007). Ligand activation of LXR beta reverses atherosclerosis and cellular cholesterol overload in mice lacking LXR alpha and apoE. *Journal of Clinical Investigation*.

[160] Quinet EM, Basso MD, Halpern AR (2009). LXR ligand lowers LDL cholesterol in primates, is lipid neutral in hamster, and reduces atherosclerosis in mouse. *Journal of Lipid Research*.

[161] Katz A, Udata C, Ott E (2009). Safety, pharmacokinetics, and pharmacodynamics of single doses of lxr-623, a novel liver X-receptor agonist, in healthy participants. *Journal of Clinical Pharmacology*.

[162] Griffett K, Solt LA, El-Gendy BE, Kamenecka TM, Burris TP (2012). A liver-selective LXR inverse agonist that suppresses hepatic steatosis. *ACS Chemical Biology*.

[163] Mencarelli A, Fiorucci S (2010). FXR an emerging therapeutic target for the treatment of atherosclerosis. *Journal of Cellular and Molecular Medicine*.

[164] Hambruch E, Miyazaki-Anzai S, Hahn U (2012). Synthetic farnesoid X receptor agonists induce high-density lipoprotein-mediated transhepatic cholesterol efflux in mice and monkeys and prevent atherosclerosis in cholesteryl ester transfer protein transgenic low-density lipoprotein receptor (-/-) mice. *Journal of Pharmacology and Experimental Therapeutics*.

[165] Fiorucci S, Cipriani S, Baldelli F, Mencarelli A (2010). Bile acid-activated receptors in the treatment of dyslipidemia and related disorders. *Progress in Lipid Research*.

[166] Al-Allaf FA, Coutelle C, Waddington SN, David AL, Harbottle R, Themis M (2010). LDLR-Gene therapy for familial hypercholesterolaemia: problems, progress, and perspectives. *International Archives of Medicine*.

[167] Grossman M, Rader DJ, Muller DWM (1995). A pilot study of ex vivo gene therapy for homozygous familial hypercholesterolaemia. *Nature Medicine*.

[168] Plump AS, Scott CJ, Breslow JL (1994). Human apolipoprotein A-I gene expression increases high density lipoprotein and suppresses atherosclerosis in the apolipoprotein E-deficient mouse. *Proceedings of the National Academy of Sciences of the United States of America*.

[169] Maeda N, Li H, Lee D, Oliver P, Quarfordt SH, Osada J (1994). Targeted disruption of the apolipoprotein C-III gene in mice results in hypotriglyceridemia and protection from postprandial hypertriglyceridemia. *Journal of Biological Chemistry*.

[170] Gaudet D, de Wal J, Tremblay K (2010). Review of the clinical development of alipogene tiparvovec gene therapy for lipoprotein lipase deficiency. *Atherosclerosis Supplements*.

[171] Gaudet D, Méthot J, Déry S (2013). Efficacy and long-term safety of alipogene tiparvovec (AAV1-LPL(S447X)) gene therapy for lipoprotein lipase deficiency: an open-label trial. *Gene Therapy*.

[172] Marquart TJ, Allen RM, Ory DS, Baldán A (2010). miR-33 links SREBP-2 induction to repression of sterol transporters. *Proceedings of the National Academy of Sciences of the United States of America*.

[173] Horie T, Ono K, Horiguchi M (2010). MicroRNA-33 encoded by an intron of sterol regulatory element-binding protein 2 (Srebp2) regulates HDL in vivo. *Proceedings of the National Academy of Sciences of the United States of America*.

